# Targeting the MYCN-MDM2 pathways for cancer therapy: Are they druggable?

**DOI:** 10.1016/j.gendis.2023.101156

**Published:** 2023-10-27

**Authors:** Wei Wang, Yi Du, Sayantap Datta, Josef F. Fowler, Hannah T. Sang, Najah Albadari, Wei Li, Jennifer Foster, Ruiwen Zhang

**Affiliations:** a Department of Pharmacological and Pharmaceutical Sciences, College of Pharmacy, University of Houston, Houston, TX 77204, USA; b Drug Discovery Institute, University of Houston, Houston, TX 77204, USA; c College of Pharmacy, The University of Tennessee Health Science Center, Memphis, TN 38163, USA; d Texas Children's Hospital, Department of Pediatrics, Section of Hematology-Oncology Baylor College of Medicine, Houston, TX 77030, USA

**Keywords:** MDM2, MYCN, Neuroblastoma, Signaling pathway, Targeted therapy

## Abstract

Targeting oncogenes and their interactive partners is an effective approach to developing novel targeted therapies for cancer and other chronic diseases. We and others have long suggested the MDM2 oncogene being an excellent target for cancer therapy, based on its p53-dependent and -independent oncogenic activities in a variety of cancers. The MYC family proteins are transcription factors that also regulate diverse biological functions. Dysregulation of MYC, such as amplification of *MYCN*, is associated with tumorigenesis, especially for neuroblastoma. Although the general survival rate of neuroblastoma patients has significantly improved over the past few decades, high-risk neuroblastoma still presents a poor prognosis. Therefore, innovative and more potent therapeutic strategies are needed to eradicate these aggressive neoplasms. This review focuses on the oncogenic properties of *MYCN* and its molecular regulation and summarizes the major therapeutic strategies being developed based on preclinical findings. We also highlight the potential benefits of targeting both the MYCN and MDM2 oncogenes, providing preclinical evidence of the efficacy and safety of this approach. In conclusion, the development of effective small molecules that inhibit both MYCN and MDM2 represents a promising new strategy for the treatment of neuroblastoma and other cancers.

## Introduction

The MYC family comprises three prominent members, MYC (c-Myc), L-MYC, and MYCN (N-Myc), pivotal transcription factors orchestrating a myriad of cellular processes.^1^^,^^2^ The MYC proteins contain bHLH (basic helix-loop-helix) and leucine zipper motifs for DNA binding and dimerization with another transcription factor, Myc-associated factor X (MAX).^1^^,^^2^ The dimers formed between MYC proteins and MAX bind to a consensus sequence, the enhancer-box, to regulate specific gene expression under a variety of physiological and pathological conditions.^3^^,^^4^

**Figure 1:**
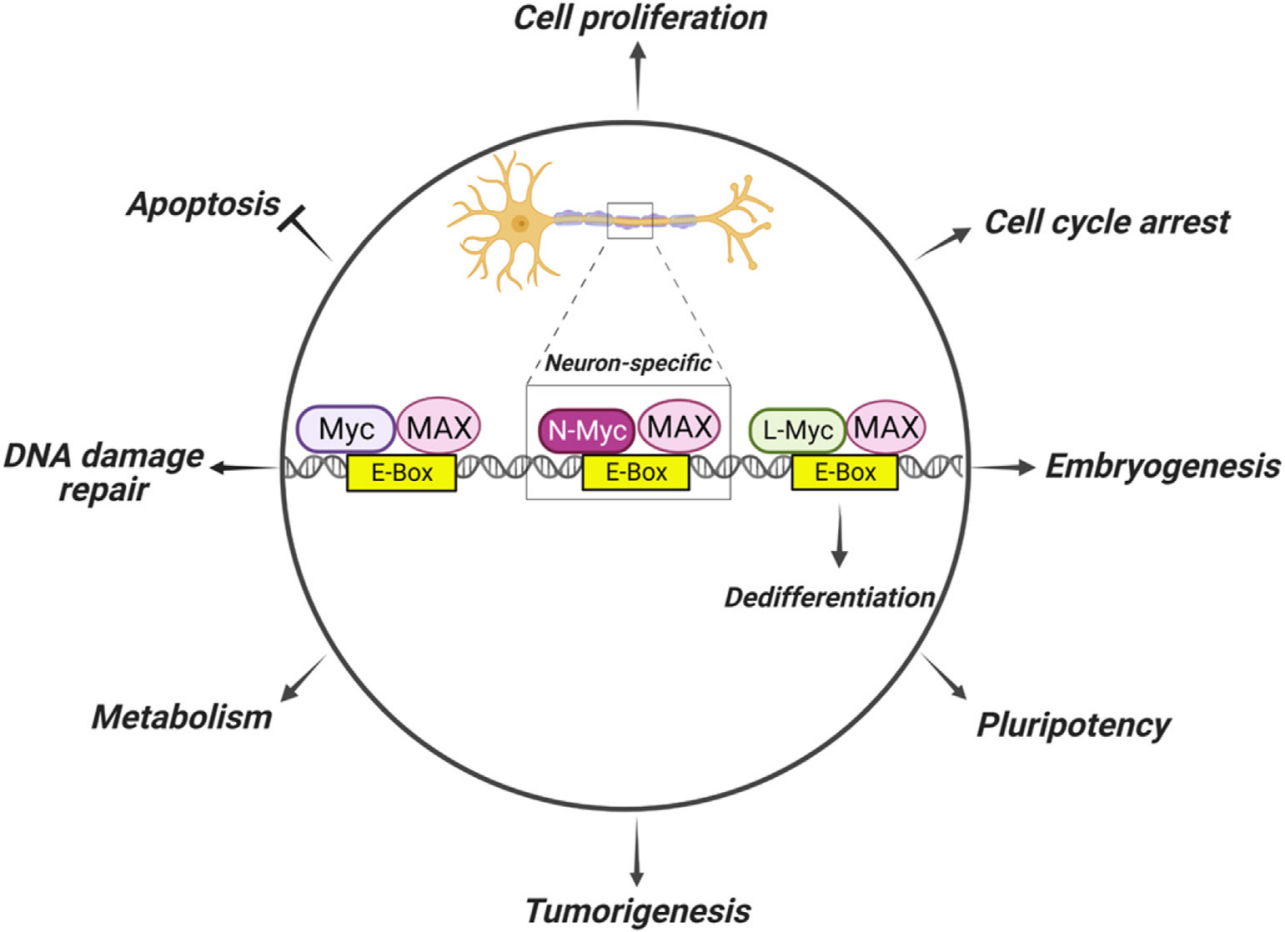
Oncogenic transcriptional regulation of the MYC family. The Myc family of transcription factors, including Myc, N-Myc, and L-Myc, bind DNA at specific sequences and regulate gene expression by binding to enhancer-box (E-Box) sequences via dimerization with Myc-associated factor X (MAX). Downstream gene expression contributes to oncogenic functions by promoting proliferation, inhibiting apoptosis, stimulating pluripotency, promoting embryogenesis, leading to abnormal metabolic regulation, increasing DNA damage repair to bypass cell death, and reducing differentiation, all of which result in more aggressive tumor growth and/or a drug-resistant phenotype.

MYC transcription factors exert a profound influence over cellular processes, encompassing proliferation, differentiation, and apoptosis.^1^^,^^2^ Perturbations in their regulatory circuits have been linked to numerous malignancies. Dysregulated MYC proteins contribute to tumorigenesis as proto-oncogenes^5^ (Fig. 1). The human c-MYC gene is located on chromosome 8 (8q24.21),^6^ comprises three exons, and belongs to one of the Yamanaka factors used to regulate the pluripotency of stem cells.^7^ The aberrant elevation of c-Myc levels is a recurring theme across diverse cancer types, fueling uncontrolled proliferation, evading cell death mechanisms, and fostering a microenvironment conducive to tumor expansion.^1^^,^^2^^,^^8^^,^^9^ MYCL was initially identified from small-cell lung cancer.^10^ It is positioned on chromosome 1 (1p34.2)^11^ and possesses five exons, and its expression exhibits selectivity, prominently observed in the gastrointestinal tract and dendritic cells.^12^^,^^13^ However, its intricate regulatory networks and dualistic functions necessitate comprehensive exploration. Another family member, MYCN, is situated on chromosome 2 (2p24.3)^14^ and composes four exons, and its expression is particularly high during the initial phases of embryonic development, primarily in nervous system cells and hematopoietic stem cells.^15^^,^^16^ While MYC is extensively expressed in neural crest stem cells, MYCN is mainly expressed in adjacent neural precursors as a part of the central nervous system during early neural development. However, MYCN knockout mice showed embryonic lethality with abnormal development of several visceral organs and peripheral and central nervous systems.^17^ This suggested that MYCN plays a larger role than initially thought. Following the discovery of the MYCN gene and amplification of MYCN in neuroblastoma,^18^, ^19^, ^20^ MYCN amplification was considered the signature for neuroblastoma, even though only around 20% of neuroblastomas carry amplification of MYCN. Dysregulation of the MYCN gene correlates with the development of various other cancer types, such as breast cancer, small-cell lung cancer, prostate cancer, basal cell carcinoma, acute lymphoblastic leukemia, and glioblastoma.^21^ In addition to the direct oncogenic functions of MYCN that arise via its regulation of gene expression, it also affects the tumor microenvironment via cytokine-mediated interactions between immune cells and tumor cells.^22^^,^^23^

Efforts to develop targeted therapies against MYC family members have predominantly focused on c-Myc due to its prominence and implication in diverse cancers. Current c-Myc inhibitors aim to perturb protein–protein interactions, modulate transcriptional activity, or induce protein degradation.^8^^,^^9^ Their demonstrated evident promise in preclinical settings underscores their significance as promising candidates for therapeutic development. Interestingly, the potential of Myc inhibitors initially designed to target c-Myc for effectively targeting MYCN is a subject of active investigation. While these inhibitors have shown promise in preclinical studies and early clinical trials, their specific effectiveness against MYCN is currently undergoing rigorous examination. Researchers are actively engaged in exploring the feasibility of repurposing c-Myc inhibitors, including bromodomain and extra-terminal domain (BET) inhibitors like JQ1, to target MYCN. Some preclinical studies have demonstrated encouraging results in inhibiting MYCN activity in MYCN-driven cancers. However, extending the utility of c-Myc inhibitors to target MYCN presents a challenge due to differing functional contexts and interactions. Despite sharing sequence homology, c-Myc and MYCN exhibit divergent functional roles due to distinct cellular contexts and interactions. These disparities are further underscored by structural differences, particularly in the N-terminal region of their protein structures. These structural distinctions can lead to variations in binding affinities and responses to inhibitors. The success of c-Myc inhibitors against MYCN hinges on the conservation of critical interfaces, necessitating rigorous investigation to determine their efficacy in this context. Consequently, emerging research is exploring tailored inhibitors specifically designed to disrupt MYCN, reflecting a paradigm shift toward precision oncology strategies. Due to its pivotal involvement in tumorigenesis, various strategies have been employed to target MYCN. These approaches aim to interfere with MYCN expression, induce protein instability, target MYCN cofactors, diminish downstream gene expression regulation, or employ a degradation approach to directly target MYCN.^21^^,^^24^ For example, inhibitors targeting BET have been tested in clinical trials and may represent an effective approach to treating MYCN-driven tumors.^25^ Further investigations of the mechanism(s) underlying MYCN-driven tumor development can shed light on the regulation of MYCN and its specific upstream and downstream signaling pathways, which will provide more opportunities to develop therapeutic strategies for MYCN-driven cancer.^26^

MDM2 is one of the most frequently studied oncogenes.^27^, ^28^, ^29^, ^30^, ^31^, ^32^ It was first discovered as a crucial regulator of the tumor suppressor p53.^33^^,^^34^ Various studies, including our own, have established that MDM2 possesses both p53-dependent and -independent oncogenic activities^32^^,^^35^, ^36^, ^37^, ^38^, ^39^, ^40^, ^41^, ^42^, ^43^ and thus represents a promising target for cancer therapy and other chronic diseases,^44^, ^45^, ^46^, ^47^, ^48^, ^49^, ^50^, ^51^, ^52^, ^53^ with many MDM2 inhibitors currently in preclinical and clinical development.^28^^,^^29^^,^^51^^,^^54^, ^55^, ^56^, ^57^, ^58^, ^59^, ^60^, ^61^, ^62^, ^63^, ^64^ Interestingly, MYCN was reported to regulate the expression of MDM2,^65^ and MDM2's oncogenic functions may contribute to MYCN-driven tumorigenesis.^66^, ^67^, ^68^, ^69^, ^70^ Targeting both MYCN and MDM2 by combining treatments or using dual inhibitors may hold promise as a potential approach for cancer patients with MYCN amplification.

Ongoing research is continuously uncovering the complexities of MYCN's functions and its potential as a therapeutic target. Gaining insights into MYCN's biology and its regulation is crucial for the development of targeted therapies and the enhancement of the prognosis for cancer patients displaying MYCN overexpression. While MYCN overexpression is observed in numerous cancer types, this review aims to consolidate the latest findings regarding MYCN biology. It covers topics such as downstream gene expression, the regulation of MYCN gene expression and protein stability, and its oncogenic roles across various cancer types, with a particular emphasis on neuroblastoma. We also describe the current status of treatments targeting MYCN in preclinical and clinical studies. In addition, we discuss the crosstalk of MYCN with other signaling pathways, such as the MDM2/p53 axis. Finally, we describe potential therapeutic approaches targeting both MYCN and MDM2 for the treatment of MYCN-driven cancer. Our discussion on this topic can contribute to the facilitation of discovering and developing innovative therapies for high-risk neuroblastoma, and the principles presented may also be applied to other cancer types.

## The role of MYCN during tumorigenesis and cancer progression

MYCN plays a critical role as a transcription factor during early embryonic development, especially in the nervous system, where it helps to maintain neuronal progenitor cells.^71^ The expression of MYCN during the gastrulation stage of mouse embryonic development suggested that it plays important roles in central or peripheral nervous system development.^72^ This was confirmed by the detection of MYCN expression in other developed organs, such as the cranial and spinal ganglia, heart, lungs, kidneys, and gut.^73^ This discovery of MYCN's involvement in embryogenesis explains why dysregulated MYCN is linked to some embryonic signatures in MYCN-driven cancers.

The discovery of MYCN overexpression in neuroblastoma cells has guided the majority of studies regarding the oncogenic functions of MYCN.^18^^,^^19^ Neuroblastoma, which originates from the peripheral nervous system neural crest cells, is responsible for around 15% of pediatric cancer-associated mortality. Amplified MYCN has been detected in approximately 20% of all neuroblastomas, and is present in about 40% of high-risk neuroblastomas.^74^ Transgenic mice with tyrosine hydroxylase promoter-driven expression of MYCN in the neural crest developed neuroblastoma, indicating that MYCN amplification alone is sufficient to induce the development of this cancer. Conditional expression of MYCN in the neural crest increased the incidence of medulloblastoma and neuroblastoma, confirming the oncogenic role of MYCN.^75^

In embryonal malignancies such as neuroblastoma and other childhood central nervous system tumors, MYCN appears to promote tumor development by utilizing pathways similar to those by which MYCN maintains progenitor cells during normal embryonic development^76^ (Table 1). Functional investigations revealed that MYCN expression is strictly regulated under physiological conditions, and its expression is required to maintain the properties of stem cells or cancer stem cells.^77^ Ectopic expression of MYCN has been shown to enhance neurosphere formation in neural crest cells, a hallmark of stem cells, and to promote symmetrical cell division, which is associated with self-renewal in human neuroblastoma cells.^78^ Results from an investigation of somatic cells with MYCN expression to promote reprogramming into pluripotency show that MYCN not only maintains the status of progenitor cells during embryonic development but also promotes oncogenic functions by increasing the self-renewal capacity and pluripotency of cells.^74^ When endogenous MYCN is deleted in induced pluripotent stem cells and embryonic stem cells, their pluripotency, self-renewal capacity, and survival are limited, leading to the induction of differentiation. Embryonic stem cells isolated from MYC- or MYCN-knockout mice also exhibit reduced self-renewal and pluripotency. However, ectopic re-expression of MYC or MYCN leads to the recovery of stem cell pluripotency,^79^ supporting the roles of both MYC and MYCN in maintaining the pluripotency of embryonic stem cells.

**Table 1:** Amplification and overexpression of MYCN in human cancers.

Cancer type	Amplification or overexpression
Neuroblastomas	20% of all neuroblastomas and 40% of high-risk neuroblastomas.^186^
Rhabdomyosarcoma	Half of the rhabdomyosarcoma (RMS) cell lines had MYCN expression.^187^ MYCN amplification was detected in 3/7 (42.9%) alveolar RMS samples but in none of the embryonal RMS samples.^188^ Twenty-three (20.4%) of 113 RMS samples showed high-level MYCN copy number changes; by subtyping, 12 (25%) of 48 alveolar RMS cases and nine (16%) of 58 embryonal RMS cases showed high-level MYCN copy number changes.^189^
Medulloblastoma	MYCN amplification was detected in three Sonic Hedgehog medulloblastomas.^190^ MYCN amplification was observed in only 4/77 (5.2%) tumors.^191^
Wilms tumor	MYCN was detected in patient samples by comparative genomic hybridization.^192^*MYCN* gains were detected in 12.7% of Wilms tumors and 30.4% of diffuse anaplastic Wilms tumors.^82^
Retinoblastoma	There was positive MYCN staining for 10/149 (6.7%) tumors.^193^
Hepatoblastoma	MYCN was detected in the aggressive C2 subtype.^194^
Glioblastoma	MYCN was detected in 40% of tumor samples.^195^
Prostate cancer	MYCN was present in 40% of neuroendocrine prostate cancers.^93^
Hematologic malignancies	MYCN was noted in pediatric T-cell acute lymphoblastic leukemia.^196^
Lung cancer	Six of 31 independently derived human small-cell lung cancer cell lines had 5- to 170-fold amplification of N-myc gene sequences.^197^
Pancreatic cancer	Three out of nine human pancreatic neuroendocrine tumors expressed MYCN.^198^

MYCN expression or overexpression has been associated with a variety of tumors. Retinoblastoma, a pediatric eye tumor, is commonly caused by mutation or loss of function of the tumor suppressor gene retinoblastoma. Retinoblastoma protein inactivation leads to MYCN overexpression, resulting in retinoblastoma tumorigenesis and up-regulation of genes that promote the proliferation of retinoblastoma cells in mice.^80^ Hepatoblastoma is a common pediatric liver malignancy. A subtype of hepatoblastoma showed embryonic properties, along with a poorer prognosis and a more advanced-stage presentation. Higher expression of MYCN has been detected in this sub-population of hepatoblastoma patients^81^**.** Wilms tumor is a pediatric kidney cancer that develops from renal stem cells with embryonic capacity and also exhibits alterations in MYCN,^82^ but abnormal metabolic regulation has been considered the major contributor to its development.^83^ However, the role of active MYCN in regulating metabolic enzymes and maintaining the “stemness” of cells in Wilms tumor is still unknown.

MYCN overexpression and amplification are frequently associated with glioblastoma multiforme and are documented in approximately 40% of tumor samples.^84^ A subtype of pediatric glioma carrying an H3.3 G34 mutation has up-regulated MYCN expression, and an aggressive malignant type of spinal ependymoma in both children and adults correlates with MYCN amplification.^85^ The MYCN amplification status may be used to categorize another subtype of glioma in addition to H3 or IDH1 mutations. Targeting MYCN with inhibitors that block the Aurora-A/MYCN complex or BET domain showed anticancer effects against glioblastoma multiforme in cell-based assays.^86^

The overexpression of MYCN is not limited to gliomas, including astrocytoma, meningioma, and glioblastoma, but is also detected in various other cancer types, including prostate cancer, hematological malignancies, lung cancer, and pancreatic cancer.^21^ Transplantation of bone marrow cells with ectopic expression of MYCN has been shown to induce the development of acute myeloid leukemia in mice,^87^ further supporting the oncogenic role of MYCN and suggesting that MYCN may also play a role in cancers involving myeloid cells. In lung cancer, even though MYCL is the major MYC family member detected, MYCN was confirmed to regulate the chemoresistance of small-cell lung cancer and promote the proliferation of non-small-cell lung cancer.^88^, ^89^, ^90^ Neuroendocrine prostate cancer (NEPC), comprising approximately 2% of all prostate cancers,^91^ is commonly castration-resistant and is characterized by the down-regulation of the androgen receptor and prostate-specific antigen expression, which are associated with a poorer prognosis.^92^ Notably, MYCN is overexpressed and amplified in around 40 % of NEPC, and this correlates with mutations or deletions of retinoblastoma protein 1 and TP53.^93^ During treatments targeting the androgen receptor, MYCN promotes changes of cancer origin from epithelial to neuroendocrine, which suggests that MYCN can contribute to the NEPC phenotype and drug resistance of prostate cancer.^94^

MYCN expression correlates with the clinical stage and outcome of patients with breast cancer.^95^ Additionally, increased MYCN has been associated with the metastatic properties of breast cancer.^96^ A subtype of serous ovarian cancer was recently identified through database analysis, and one of the associated genes identified was overexpressed MYCN.^97^ High-grade serous ovarian carcinoma with high expression of MYCN was sensitive to a BET inhibitor, suggesting that MCYN may be one of the drivers of the development of high-grade serous ovarian carcinoma.^98^

The research to date suggests that MYCN not only plays roles in the development and progression of neuronal cancer types, but dysregulated MYCN is also associated with many other types of cancer and may be related to the development and progression of those cancers.

## Regulation of MYCN expression

**Figure 2:**
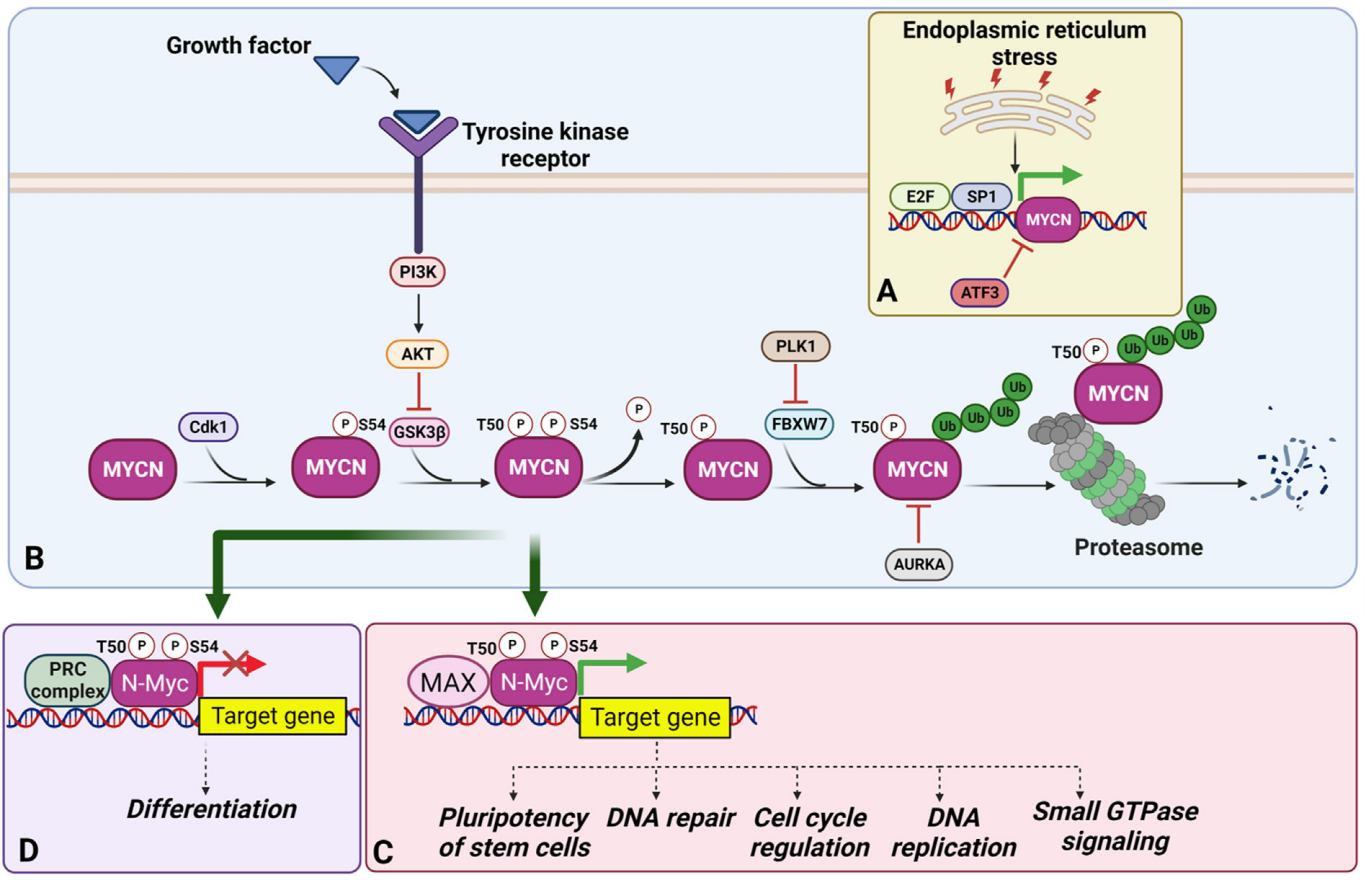
Regulation of MYCN. **(A)** Transcription factors such as E2 promoter binding factor (E2F) and specific protein 1 (SP1) can directly increase the expression of MYCN while activating transcription factor 3 (ATF3) reduces MYCN expression. **(B)** Several kinases activate the transcriptional function of MYCN either by directly phosphorylating the protein or via down-regulation of an E3 ligase to affect the protein stability of MYCN. **(C)** Phosphorylated MYCN regulates downstream gene expression and further promotes tumorigenesis. **(D)** MYCN associates with the polycomb repressive complex (PRC) to suppress the expression of genes required for cell differentiation. AKT, protein kinase B; AURKA, aurora kinase A; FBXW7, F-Box and WD repeat domain containing 7; GSK-3β, glycogen synthase kinase 3 beta; MAX, Myc-associated factor X; PI3K, phosphoinositide 3-kinase; PLK1, polo-like kinase-1.

MYCN expression is regulated by several transcription factors, including specific protein 1 (SP1)^99^ and E2 promoter binding factor.^100^ However, the transcriptional repressor, activating transcription factor 3, negatively regulates MYCN expression in response to endoplasmic reticulum stress caused by lipid desaturation in liver cancer cells^101^ (Fig. 2A).

Apart from direct regulation by transcriptional regulators, MYCN activity can also be controlled by modifying its stability and function. Glycogen synthase kinase 3 (GSK3)^102^ and Cdk1^103^ respond to phosphorylation (of threonine 50 and serine 54, respectively) to stabilize MYCN protein levels. TRIM32, an E3 ligase, promotes the degradation of MYCN via the proteasome at specific spindle poles to promote asymmetrical cell division, suggesting that TRIM32 functions as a negative regulator of MYCN to restrict MYCN's self–renewal properties.^104^ Phosphorylated MYCN recruits another E3 ligase, F-Box, and WD repeat domain containing 7 (FBXW7), which leads to the ubiquitination and subsequent degradation of MYCN. In contrast, polo-like kinase-1 phosphorylates FBXW7 and promotes its auto-polyubiquitination and degradation, resulting in MYCN stabilization^105^ (Fig. 2B). Similarly, aurora kinase A (AURKA) facilitates the interaction between N-Myc and FBXW7 and blocks FBXW7-mediated degradation of MYCN to reduce growth signals.^106^ Forkhead box protein R2, another transcription factor, interacts with MYCN and shows a positive correlation with MYCN expression, suggesting that it regulates MYCN and that the forkhead box protein R2-MYCN complex may affect the transcription of other genes.^107^ It was reported that a histone protein (H3F3A) with a G34 mutation could epigenetically up-regulate the expression of MYCN.^85^ In addition, the G34 mutant of H3F3A promotes the function of polycomb repressive complex 2,^108^ linking EZH2 and MYCN.^109^

## Gene expression mediated by MYCN

As a transcription factor, MCYN functions similarly to its family member, MYC, by forming a dimer with MAX and binding to a consensus CACGTG sequence to mediate the gene expression of downstream targets.^110^ MYCN has been shown to induce several genes associated with pluripotency in neural stem cells, such as kruppel-like factor 2/4 and Lin-28 homolog B, through its binding to their promoters as revealed by ChIP sequencing analysis in neuroblastoma (Fig. 2C).^111^ This may explain how MYCN functions as a key regulator during early development and implies that the oncogenic functions of MYCN during tumor development may be due to its maintenance of cancer stem cells. Additionally, a ChIP-on-chip analysis has identified 157 target genes that were regulated by MYCN, including those involved in DNA repair, DNA replication, cell cycle progression, and small GTPase signaling, all of which are important pathways in tumorigenesis.^112^

In addition to up-regulating gene expression, MYCN is also involved in suppressing gene expression via the polycomb repressive complex. ChIP-on-chip and MeDIP analyses have shown that the MYCN binding site significantly correlates with DNA hypermethylation, suggesting that MYCN may have suppressive effects on some downstream gene targets.^113^ Mechanistically, it has been demonstrated that MYCN-mediated suppressive regulation of gene expression involves the recruitment of the polycomb repressive complex 1 and ubiquitination of histone 2A at lysine 119^114^ (Fig. 2D). Several individual MYCN target genes have been identified. For example, multidrug resistance-associated protein 1, a member of the ABC family of transporters, is known to be associated with drug resistance and has been identified as a downstream transcriptional target of MYCN in neuroblastoma.^115^ The expression of another multidrug resistance gene, MRP4, also correlates with MYCN amplification in neuroblastoma.^116^ Eukaryotic translation initiation factor 4E-binding protein 1, a repressor of transcription, is up-regulated by MYCN and is associated with a poor prognosis in neuroblastoma.^117^ MYCN has also been shown to transcriptionally up-regulate p53 in neuroblastoma, which in turn increases the levels of mouse double minute 2 (MDM2) and p53 up-regulated modulator of apoptosis.^118^

## Positive feedback regulation of MYCN

**Figure 3:**
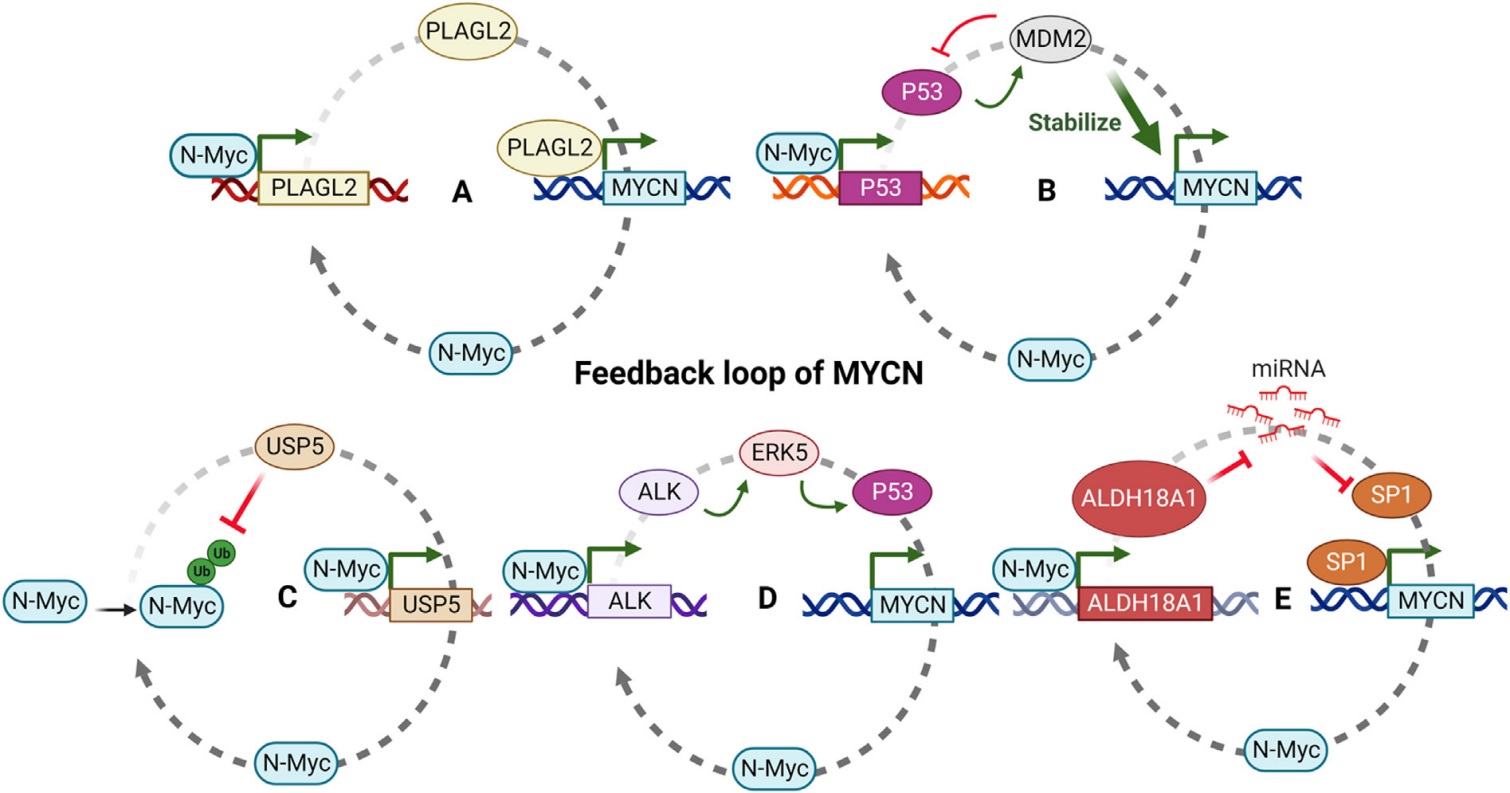
Feedback regulation of MYCN. MYCN and its target genes form several feedback loops, playing important roles in regulating MYCN expression and function in tumorigenesis. **(A)** Pleiomorphic adenoma gene-like 2 (PLAGL2) is a transcription factor that directly activates the transcription of MYCN. MYCN can also activate PLAGL2 expression, forming a positive feedback loop. **(B)** MYCN upregulates p53, which leads to increased mouse double minute 2 (MDM2) expression and MDM2 binds to the mRNA of MYCN, stabilizes, and enhances its translation. **(C)** Ubiquitin-specific peptidase 5 (USP5) is a deubiquitinase that specifically deubiquitinates and stabilizes MYCN, forming a positive feedback loop. **(D)** Anaplastic lymphoma kinase (ALK) is a receptor tyrosine kinase that functions as an upstream signaling molecule to regulate MYCN expression. MYCN can also promote ALK expression, forming a positive feedback loop. **(E)** Aldehyde dehydrogenase 18 family member A1 (ALDH18A1) decreases the miRNA expression of both specific protein 1 (SP1) and MYCN, forming a negative feedback loop. These feedback loops highlight the complex and dynamic regulation of MYCN expression and activity in cancer cells. ERK5, extracellular signal-regulated protein kinase 5.

Positive feedback loops involving MYCN and its partner proteins highlight the intricate regulation of MYCN activity (Fig. 3). Pleiomorphic adenoma gene-like 2, a zinc finger protein, binds to the promoter region of MYCN and is involved in its gene expression.^119^ In turn, MYCN also acts as a transcription factor for it, establishing a positive feedback loop (Fig. 3A). Similarly, MDM2 binds to the mRNA of MYCN to enhance its translation, while MYCN regulates the gene expression of MDM2, thereby maintaining the activation of both MYCN and MDM2.^69^ As mentioned earlier, MYCN up-regulates p53, leading to increased MDM2 expression, further reinforcing the MDM2/MYCN feedback loop (Fig. 3B). Ubiquitin-specific proteases, such as ubiquitin-specific peptidase 5, can block E3 ligase-mediated degradation of MYCN and stabilize the MYCN protein. MYCN, in turn, binds to the promoter of ubiquitin-specific peptidase 5 to promote its gene expression, establishing a positive feedback loop that sustains the functions of MYCN/ubiquitin-specific peptidase 5^120^ (Fig. 3C). Anaplastic lymphoma kinase (ALK) is another transcriptional target of MYCN, and ALK triggers MYCN transcription in neuroblastoma cell lines, which generates a positive feedback loop.^121^, ^122^, ^123^ Mechanistically, ALK drives MYCN expression by activating the p53 promoter via extracellular signal-regulated protein kinase 5^124^ (Fig. 3D). AURKB is also a direct transcriptional target of MYCN. The expression of AURKA/B correlates with a poorer prognosis in neuroblastoma patients, and both are considered candidates for targeting with specific inhibitors.^125^ Inhibition of the aldehyde dehydrogenase 18 family member A1-MYCN positive feedback loop attenuates the growth of MYCN-amplified neuroblastoma. Aldehyde dehydrogenase 18 family member A1 coordinates with *miR-29b*/SP1 to promote the transcription of MYCN by stabilizing MYCN mRNA^126^ (Fig. 3E).

## The crosstalk between MYCN and MDM2/p53

Similar to MDM2, which was originally discovered from double minute chromosomes in NIH-3T3 cells, MYCN was also found to form extrachromosomal double minutes in neuroblastomas. As previously mentioned, MYCN modulates the expression of MDM2 and contributes to MYCN-driven neuroblastoma.^65^ MDM2 is also a co-activator for the translation of MYCN. Cytoplasmic MDM2 can bind to the AU-rich elements of the MYCN 3′-UTR and regulate MYCN mRNA stability and translation.^127^ This mechanism was initially detected in retinoblastoma cells, where MDM2 up-regulated the mRNA expression and translation of MYCN. Overexpression of MYCN reversed the effects of MDM2 depletion, suggesting that MYCN could be regulated by MDM2.^128^ In MYCN-amplified neuroblastoma, MDM2 fosters tumor growth independently of p53. MDM2 overexpression enhances MYCN expression without affecting p53, as MYCN up-regulation stimulates p53 transcription.^69^ Conversely, silencing MDM2 does not alter p53 but reduces MYCN, diminishing p53 transcription, although MDM2's p53 degradation is reduced.^69^ Notably, enforced MDM2 overexpression or its inhibition has opposing effects on tumor growth in MYCN-amplified neuroblastoma, independent of p53 functionality.^69^ These findings suggest that p53, reciprocally regulated by MDM2 and MYCN, is not essential for suppressing MYCN-amplified neuroblastoma. Instead, the direct MDM2-MYCN interaction significantly fuels MYCN-amplified neuroblastoma growth and progression. Interestingly, studies have shown that neuroblastoma cells derived from MYCN transgenic mice with TP53 genetic mutations exhibit reduced sensitivity to MDM2 inhibitors (MI-63 and RG7388) compared with TP53 wild-type human neuroblastoma cells.^72^ This suggests that MYCN-mediated up-regulation of MDM2 may induce resistance against MDM2 inhibitors, particularly in the absence of functional p53.

Amplification of both MYCN and MDM2 has also been detected in alveolar rhabdomyosarcoma, a subtype of rhabdomyosarcoma, and is associated with the deletion of retinoblastoma protein 1.^129^ Elevated MDM2 expression is required for high-level expression of MYCN in small-cell lung cancer, retinoblastoma, neuroblastoma, and medulloblastoma cells.^69^^,^^128^^,^^130^ Notably, the ectopic expression of MYCN induces MDM2 expression, leading to enhanced proliferation of cone precursor-derived masses in a retinoblastoma genesis model under culture conditions.^130^ This crosstalk and positive feedback between MDM2 and MYCN suggest that targeting either molecule alone may not be sufficient to achieve long-term anticancer effects. The evolving landscape of transcription factor targeting, with a specific focus on MYCN and the MDM2/p53 axis, holds great promise for the treatment of various cancers, notably neuroblastoma.

## Targeted therapies for neuroblastoma

In this review, our primary focus will be on the roles of MYCN and MDM2 in the context of neuroblastoma. We will explore the intricate relationship between these two key players and their implications for the development, progression, and potential therapeutic interventions in neuroblastoma. A comprehensive comprehension of the molecular basis of neuroblastoma is essential for the development of effective therapeutic strategies and to improve morbidity and mortality.^70^ However, the survival rate of patients suffering from relapsed or refractory high-risk neuroblastoma was currently less than 15%^131^ and was even lower among patients with MYCN amplification. Aggressive multimodal therapy, which includes radiation therapy, autologous stem cell transplantation, immunotherapy, and retinoids, has increased the survival rate of patients with high-risk neuroblastoma to 50%.^132^

## Strategies currently used for the treatment of neuroblastoma

The standard treatment for high-risk neuroblastoma involves three phases: induction, consolidation, and post-consolidation or maintenance therapy. Induction therapy typically consists of chemotherapeutic agents such as vincristine, doxorubicin, cyclophosphamide, topotecan cisplatin, and etoposide, which are administered for 5–8 cycles. Surgery is another major approach typically performed near the end of induction chemotherapy after the tumor shrinks to reduce surgical morbidity. During the consolidation stage, a combination of high-dose chemotherapy, followed by autologous stem cell transplant and radiation therapy, is commonly employed. The maintenance phase of therapy aims to treat residual disease to prevent disease relapse. This phase includes anti-disialoganglioside (anti-GD2) immunotherapy and the use of differentiating agent isotretinoin^133^.

The immune response is crucial in fighting neuroblastoma. Leukocytes, especially lymphocytes, from neuroblastoma patients can inhibit tumor growth and are cytotoxic to neuroblastoma cells.^134^ Infant neuroblastoma's high leukocyte counts and spontaneous regression suggest enhancing antitumor immunity as a therapeutic approach.^135^ Recent studies reinforce this immune response and its treatment implications.^136^^,^^137^ Monoclonal antibodies targeting GD2 are approved for second-line therapy in high-risk neuroblastoma. Agents like dinutuximab, dinutuximab-beta, and naxitimab, combined with granulocyte-macrophage colony-stimulating factor and 13-cis-retinoic acid, are used post-consolidation,^138^ and with temozolomide and irinotecan for relapsed or refractory cases.^139^ Recent studies have highlighted the importance of natural killer cells in the immune response to neuroblastoma.^140^, ^141^, ^142^ Neuroblastoma cells can create an immunosuppressive microenvironment by reducing human leukocyte antigen and adhesion molecules, hindering the binding of cytotoxic T cells and natural killer cells.^143^ Restoring antitumor immunity is crucial to curbing tumor growth. Tailoring the immune response with agents that activate specific cell subsets holds promise. Chimeric antigen receptor-T-cell therapy is another promising targeted therapy for high-risk neuroblastoma treatment. Chimeric antigen receptor-T cells can be engineered to recognize and target tumor cell surface antigens like GD2, activating cytotoxic T cells to eliminate cancer cells.^144^

Genetic screening and mechanism studies have identified potential targets for preclinical exploration in high-risk neuroblastoma. In high-risk neuroblastoma, ALK abnormalities (seen in 10%–14% of cases) drive disease initiation or progression.^145^^,^^146^ Specific mutations like R1275Q, F1174L, and F1245C activate ALK,^145^ but not all respond to current inhibitors.^147^ These ALK changes are connected to MYCN amplification, forming a positive feedback loop that activates MYCN transcription in neuroblastoma cell lines.^121^, ^122^, ^123^ ALK abnormalities activate downstream pathways, including the phosphoinositide 3-kinase/protein kinase B/mTOR pathway^148^ and Ras/mitogen-activated protein kinase signal transduction pathways.^149^ Targeting the phosphoinositide 3-kinase/protein kinase B/mTOR pathway can benefit patients with high-risk neuroblastoma by inhibiting cell growth, proliferation, metastasis, and oncogenic glucose metabolism in neuroblastoma cells.^150^

Metastatic neuroblastoma relies on angiogenesis for growth and survival, driven by the expression of vascular endothelial growth factor and its receptor.^151^ Targeting angiogenesis with bevacizumab, an anti-vascular endothelial growth factor antibody, is a potential treatment strategy. Additionally, tyrosine kinase inhibitors like ponatinib and imatinib, which inhibit various growth factor receptors involved in angiogenesis, such as fibroblast growth factor receptor 1–4, rearranged during transfection, platelet-derived growth factor receptor alpha, receptor tyrosine kinase, Fms related receptor tyrosine kinase 3, mitogen-activated protein kinase kinase kinase 2, and vascular endothelial growth factor receptor 1/2, show promise in neuroblastoma therapy. Ongoing research aims to assess their effectiveness and safety in disrupting neuroblastoma progression.^152^

## Targeting MYCN and MDM2 in neuroblastoma

Amplification of MYCN is a hallmark of neuroblastoma and has been the target of several therapeutic strategies. Like many other transcription factors, achieving selective inhibition of MYCN has traditionally been considered a challenging endeavor. Moreover, the structural composition of MYCN predominantly consists of α helices, offering limited surfaces for direct ligand binding.^153^ However, despite these challenges, several proposed strategies exist for indirectly targeting N-Myc.

One approach involves disrupting the MYCN-MAX interaction. MYCN and MAX are essential transcription factors that form heterodimer complexes, regulating the expression of genes involved in proliferation, differentiation, and cell survival.^110^ Dysregulation of the MYCN-MAX complex is a hallmark of various cancers, making it an attractive therapeutic target. The bHLH-Zip domains of human c-MYC and MYCN share 56% similarity in their protein sequences.^154^ Considering the pronounced structural and functional resemblance in the C-terminal regions of c-MYC and MYCN, it is highly probable that molecules capable of binding to c-MYC would similarly interact with MYCN. Among them, the extensively studied small molecule 10058-F4, initially designed for c-MYC, has shown potential in targeting both c-MYC and MYCN.^155^ The compound binds to the region AA402–409 of the MYC protein, thereby disrupting the formation of MYC-MAX dimers involving either c-MYC or MYCN.^154^ This dual inhibition results in MYCN inhibition, cell cycle arrest, apoptosis induction, and neuronal differentiation promotion, particularly in MYCN-amplified neuroblastoma cells compared with their non-MYCN amplified counterparts.^155^

Additionally, these insights have been substantiated by compelling evidence demonstrating a noteworthy delay in tumor growth within the SK-N-BE (2) neuroblastoma xenograft model and an extension of survival in a *TH*-*MYCN* transgenic mouse model of neuroblastoma (established by targeted expression of the human MYCN oncogene in neuroectodermal cells under the control of rat tyrosine hydroxylase promoter).^155^ Omomyc, a compound derived from the bHLH-Zip domain of Myc, disrupts Myc homodimerization by replacing four amino acids within the Myc zipper.^156^ It can bind to c-Myc, N-Myc, Max, and Miz-1, thereby preventing Myc from binding to promoter enhancer-boxes and activating target genes while simultaneously retaining Miz-1-dependent transrepression. *In vivo* studies have demonstrated Omomyc's antitumor activity,^156^ offering insights into the potential development of clinical inhibitors targeting bHLH-ZIP proteins.

Since the BET domain in several transcriptional regulators is involved in regulating MYCN, targeting the BET bromodomain with small molecule inhibitors is expected to enhance cell death by interfering with MYCN transcription. Two BET inhibitors are currently being tested in clinical trials, including trials for neuroblastoma (NCT03936465, NCT01587703). In MYCN-amplified neuroblastoma, MYCN gene amplification drives oncogenic processes by promoting the transcription of genes necessary for tumor growth. Among these, BET proteins, particularly BRD4, are involved in this transcriptional activation.^157^ JQ1, the first developed BET inhibitor, disrupts this process by competitively binding to the bromodomain of BET proteins, preventing their interaction with acetylated histones.^158^ Preclinical studies demonstrated its effectiveness in reducing MYCN expression, inhibiting cell proliferation, and inducing apoptosis.^158^ Clinical trials are underway to further evaluate its potential as a treatment, albeit with some challenges, such as drug delivery and toxicity issues. ARV-825 is an effective BET inhibitor employing PROTAC technology to degrade target proteins via the proteasome.^159^ It demonstrates potent anticancer properties, including the suppression of proliferation, cell cycle arrest, and induction of apoptosis in neuroblastoma cells.^159^ ARV-825 also efficiently depletes BET protein levels, leading to the repression of MYCN or c-Myc expression.^159^ In a neuroblastoma xenograft model, ARV-825 significantly down-regulates BRD4 and MYCN expression and reduces tumor growth in mice.^159^ ARV825 shows significant potential as a therapeutic strategy for combating MYCN-driven cancers and other malignancies.

In addition to MYCN-targeting approaches, small molecule inhibitors can also be used to target other key molecules that affect MYCN stability in neuroblastoma. For instance, AURKA and mTOR can be targeted with small molecules to inhibit MYCN function. Upon activation, ALK facilitates the recruitment and activation of the phosphoinositide 3-kinase/protein kinase B pathway, which in turn regulates the activity of glycogen synthase kinase 3 beta and influences the stability of MYCN protein^160^,^161^. Targeting protein kinase B beta,^162^ mTOR,^163^ and ALK^164^ can inhibit MYCN function. Through direct protein–protein interaction, AURKA stabilizes MYCN, resulting in reduced degradation of MYCN by the proteasome.^106^ AURKB is involved in the feedback regulation of AURKA and the MYCN loop. Therefore, targeting AURKA/B represents a potential strategy for inhibiting MYCN-driven neuroblastoma. MLN8054 and MLN8237 are notable compounds recognized for their ability to disrupt the AURKA/N–Myc complex.^165^ They facilitate N-Myc degradation through the involvement of the Fbxw7 ubiquitin ligase.^165^ By disrupting this complex, these compounds effectively inhibit N-Myc-dependent transcription, leading to tumor regression and prolonged survival in a mouse model of MYCN-driven neuroblastoma.^165^ PROTAC SK2188 is a promising compound in targeted protein degradation.^166^ It demonstrates exceptional potency in degrading AURKA and exhibits a remarkable binding and selectivity profile.^166^ When applied to NGP neuroblastoma cells, SK2188 degrades AURKA, induces MYCN degradation, triggers replication stress and DNA damage, and leads to apoptosis.^166^ Additionally, SK2188 outperforms the parent inhibitor MK-5108 in inhibiting cell proliferation and shows superior efficacy in patient-derived organoid models, highlighting its potential as a valuable therapeutic agent.^166^ Barasertib is a well-known compound recognized for its selective inhibition of AURKB.^167^ It has demonstrated remarkable efficacy, particularly in the context of MYCN-amplified neuroblastoma. Barasertib exerts its action by disrupting AURKB activity, which leads to alterations in the phosphorylation of key proteins like histone H3 and influences crucial cell cycle processes.^167^ Its effectiveness includes the induction of cell cycle arrest, endoreduplication, and apoptosis in cancer cells.^167^ Moreover, barasertib has exhibited promising outcomes in preclinical studies and neuroblastoma xenograft models, positioning it as a potential candidate for clinical trials as a cancer therapy.^167^

Based on the transcriptional repression mediated by MYCN and the polycomb repressive complex 2 repressive complex, targeting EZH2 (the catalytic subunit of polycomb repressive complex 2), which is overexpressed in neuroblastoma cells, is another approach that might be used to inhibit the functions of MYCN.^109^ GSK343 is a small molecule inhibitor that specifically targets EZH2.^168^ GSK343, as an S-adenosylmethionine-competitive EZH2 inhibitor, inhibits EZH2 by binding to the site for S-adenosylmethionine within EZH2's binding pocket. This binding interferes with EZH2's enzymatic activity, specifically its ability to use S-adenosylmethionine as a cofactor for histone methylation, ultimately leading to the inhibition of EZH2-mediated histone modifications and gene regulation. Treatment with GSK343 led to significant benefits in neuroblastoma research, including decreased cell viability, inhibited migration and invasion, and reduced stemness in neuroblastoma patient-derived xenograft cells.^168^ Moreover, GSK343 demonstrated the potential to suppress tumor growth in mice bearing SK-N-BE (2) neuroblastoma tumors.^168^ EZH2 inhibitors like JQEZ5 and GSK126 have shown notable efficacy, particularly in MYCN-amplified cell lines.^169^ These MYCN-amplified cell lines exhibited significantly greater sensitivity to EZH2 inhibition compared with MYCN-non-amplified counterparts.^169^ This finding underscores the potential utility of EZH2 inhibitors as a targeted therapeutic approach in MYCN-driven cancers, offering a ray of hope for more effective treatments in these challenging cases. Further research is required to elucidate the optimal targets by dissecting the regulatory protein complexes that collaborate with MYCN in the oncogenic regulation of various cellular processes, such as DNA replication, transcription, splicing, and other essential functions.

The majority of neuroblastomas carry wild-type, functional p53. However, inactivation of the p53 pathway is associated with recurrence and chemoresistance. MDM2 can inactivate p53. Therefore, targeting MDM2 to restore p53 activity could be an effective approach to treating neuroblastoma.^29^ This approach has demonstrated significant potential in the treatment of neuroblastoma. Both preclinical investigations and ongoing clinical trials have assessed various MDM2 inhibitors, as comprehensively reviewed in a recent study.^28^ Among these, Nutlin-3 stands out for its ability to activate the p53 pathway, restraining primary tumor growth and metastasis in preclinical neuroblastoma models. Various MDM2 inhibitors, such as SAR405838 (MI-77301), MK-8242, MI-63, RG7388 (RO5503781), RG7112 (RO5045337), and RG7775 (RO6839921), have also exhibited potential in stabilizing p53 and inducing apoptosis in neuroblastoma cells.^28^ To be more specific, in MYCN-amplified (MNA) neuroblastoma cells, Nutlin-3 not only disrupts the interaction between p53 and MDM2 but also leads to the accumulation of p53 and homeodomain-interacting protein kinase 2, ultimately triggering programmed cell death.^170^ Furthermore, Nutlin-3 has demonstrated synergistic effects when combined with clastogenic drugs such as cisplatin, doxorubicin, and bleomycin, leading to the down-regulation of galectin-3 in MNA neuroblastoma cells.^171^ Additionally, other research has shown that the presence of MYCN amplification in neuroblastoma has a significant impact on the sensitivity to MDM2-p53 antagonists like Nutlin-3 and MI-63. When MYCN is knocked down, there is a decrease in the sensitivity of MYCN and MDM2 co-amplified neuroblastoma cells to Nutlin-3 and MI-63 treatment. This implies that MYCN amplification sensitizes these cells to the effects of MDM2-p53 antagonists.^172^ Conversely, knockdown of MYCN results in increased resistance of MYCN-amplified neuroblastoma cell lines to the induction of p53 and apoptosis by Nutlin-3 and MI-63. It is noteworthy that MYCN-amplified neuroblastoma cell lines are inherently more sensitive to the growth-inhibitory effects of MDM2-p53 antagonists compared with non-MYCN-amplified neuroblastoma cell lines.^172^ These findings underscore the complex interplay between MYCN and the MDM2-p53 axis in neuroblastoma and suggest that targeting this interaction may hold promise as a therapeutic strategy, particularly in MYCN-amplified cases.

Our lab has been discovering and developing MDM2 inhibitors for cancer therapy.^29^^,^^54^^,^^55^^,^^60^^,^^61^ Recently, we have demonstrated that SP141, a small molecule that induces MDM2 degradation and inhibits MYCN protein level, exhibits several beneficial effects in neuroblastoma.^29^ This compound reduces cell viability, promotes apoptosis, arrests cells at the G2/M phase of the cell cycle, and inhibits cell migration. Importantly, these effects are observed in both *in vitro* experiments and *in vivo* models of neuroblastoma, and they occur in a manner independent of p53.^29^ Significantly, SP141 demonstrated the ability to inhibit MDM2 expression and effectively suppress tumor growth without causing any host toxicity at the effective dosage. These findings highlight the safety profile of SP141 as a potential therapeutic agent. These proof-of-concept results strongly suggest that SP141 holds promise as a novel treatment option for neuroblastoma, regardless of the p53 status. Recently, the phase 1 study of the dual MDM2/MDMX inhibitor ALRN-6924 in pediatric cancer demonstrated safety, tolerability, and promising antitumor activity in relapsed or refractory solid tumors and acute lymphoblastic leukemia. The drug showed target engagement and p53 pathway activation, supporting its potential as a therapeutic option for pediatric cancers (NCT03654716). Notably, a natural product from *Nardostachys jatamansi* roots has been shown to simultaneously down-regulate the expression of both the MYCN and MDM2 proteins and increase the expression of p53 in neuroblastoma cells, indicating that dual targeting of MDM2 and MYCN is a possibility.^173^

In addition, combining MDM2 antagonists with other drugs or experimental compounds has the potential to enhance therapeutic outcomes and offer a more effective treatment strategy, ultimately leading to reduced relapse rates in neuroblastoma patients.^28^ The disruption of MDM2 regulation in neuroblastoma and the engagement of MYC family proteins in high-risk disease have led to the proposition that simultaneous targeting of both the MDM2 and MYCN might result in a synergistic increase in cytotoxicity in neuroblastoma models, providing a potential innovative therapeutic avenue. The combination of CGM097, an MDM2 inhibitor, and OTX015, a bromodomain inhibitor, has led to the activation of p53 and decreased expression of MYC proteins, resulting in neuroblastoma cell death.^174^ The combination of CGM097 and venetoclax has displayed remarkable effectiveness in MYCN-amplified, p53-WT neuroblastoma.^175^ This has been associated with a rapid increase in the transcription of BBC3 (p53 up-regulated modulator of apoptosis) and PMAIP1 (NOXA) shortly after p53 activation, indicating a swift response to MDM2 inhibition by NVP-CGM097.^175^ Furthermore, the combination of CGM097 with venetoclax effectively has suppressed tumor growth in MYCN-amplified neuroblastoma patient-derived xenograft models.^175^

## Targeting MYCN and MDM2 in diverse cancers

MYCN and MDM2 inhibition has gained attention not only in neuroblastoma but also in a spectrum of other cancer types due to their potential in targeting key oncogenic pathways. Common MDM2 inhibition strategies, which typically involve targeting the MDM2 protein to restore the function of the p53 tumor suppressor, have shown promise in preclinical studies and clinical trials for various types of cancer. However, the effectiveness of MDM2 inhibition can vary depending on the specific cancer type and the underlying molecular mechanisms involved.^28^^,^^29^^,^^176^, ^177^, ^178^ The intriguing prospect of employing MYCN targeting strategies as a universal approach for managing cancers with MYCN overexpression warrants discussion. Studies are ongoing to investigate the therapeutic potential of MYCN inhibitors in various cancers, including medulloblastoma, rhabdomyosarcoma, and NEPC.^24^

Medulloblastoma, a highly malignant brain tumor primarily affecting children, poses a formidable challenge in oncology.^179^ Among its subtypes, non-WNT and non-Sonic Hedgehog medulloblastoma are frequently associated with MYCN amplification, which significantly heightens their aggressiveness and worsens prognosis.^179^ In these MYCN-amplified medulloblastomas, MYCN emerges as a pivotal oncogenic driver, orchestrating uncontrolled cell growth and resistance to conventional treatments. These inhibitors have been intricately designed to selectively target the molecular abnormalities driven by MYCN amplification, sparing normal cells from unintended harm. In the realm of medulloblastoma treatment, a range of innovative strategies is under investigation. These approaches encompass stabilizing MYCN, controlling MYCN's transcriptional activity, influencing MYCN-related epigenetics, disrupting MYC-MAX complexes, and uncovering synthetic lethal targets of MYCN. These strategies represent a dynamic landscape of research aimed at advancing our understanding and treatment of medulloblastoma.^179^ MYCN inhibitors encompass a spectrum of compounds, including the BET inhibitor JQ1, HDAC inhibitor Panobinostat, and phosphoinositide 3-kinase inhibitor buparlisib, each with its distinct mechanism of action.^179^ While clinical trials are actively assessing their safety and efficacy in medulloblastoma patients, challenges persist, including tumor heterogeneity, the emergence of resistance mechanisms, and the imperative consideration of pediatric patients' unique needs. Despite these challenges, MYCN inhibitors hold promise as a crucial component in the evolving therapeutic landscape against MYCN-driven medulloblastomas, potentially ushering in a new era of more effective and less debilitating treatments for affected children.

Rhabdomyosarcoma, the most prevalent soft-tissue sarcoma among children, presents a pressing challenge in pediatric oncology.^179^ Within this cancer, genomic amplification of MYCN serves as a well-established harbinger of poor prognosis, particularly pronounced in the aggressive alveolar subtype. In response, researchers are exploring diverse therapeutic strategies to mitigate the adverse effects of MYCN amplification. These approaches encompass various fronts, including the development of small molecular inhibitors that disrupt MYC-MAX dimerization, strategies to block MYCN's transcriptional activity, the innovative use of nucleic acid-based techniques like antisense and peptide nucleic acids, and the exciting prospect of immunotherapy.^179^ Notably, MYCN's unique expression profile, predominantly absent in mature tissues but frequently dysregulated in a substantial portion of alveolar rhabdomyosarcoma cases, makes it an enticing target for immunotherapeutic interventions. In fusion-positive rhabdomyosarcoma, inhibiting AURKA disrupts MYCN, a crucial oncogene regulated by PAX3-FOXO1.^180^ When AURKA inhibitor alisertib is combined with navitoclax (a Bcl2 inhibitor) in experiments involving fusion-positive rhabdomyosarcoma cell lines and patient-derived xenografts, a potent synergy emerges. This combined treatment not only triggers substantial cell death but also markedly decelerates tumor growth in preclinical models.^180^ These findings provide fresh insights into fusion-positive rhabdomyosarcoma molecular dynamics, offering potential for innovative combination therapies. Dual polo-like kinase-1 and BRD4 inhibitor, UMB103, suppresses cell proliferation and triggers apoptosis at low nanomolar concentrations in rhabdomyosarcoma cells.^181^ Notably, this treatment also leads to a marked reduction in MYCN-driven gene expression, as evidenced by RNA sequencing.^181^ The administration of the UMB103 to patient-derived xenograft models results in substantial tumor regression.^181^ These findings underscore the effectiveness of simultaneously targeting two pivotal regulators of the MYC protein family, BRD4 and polo-like kinase-1, using single small molecules, highlighting their potent and selective antitumor capabilities in pediatric cancer models. Collectively pursued with determination and innovation, these strategies hold promise in enhancing the prospects and well-being of children confronting this formidable cancer.

Neuroendocrine prostate cancer is a highly aggressive subtype of prostate cancer that often exhibits neuroendocrine differentiation.^182^ Elevated N-Myc expression is linked to the development of aggressive prostate cancer with neuroendocrine characteristics, resembling NEPC. This transformation is marked by the suppression of androgen receptor signaling and the activation of polycomb repressive complex 2/EZH2 signaling.^182^ Concrete evidence demonstrates that NEPC can indeed originate from a common epithelial clone.^94^ Moreover, MYCN plays a pivotal role in maintaining NEPC, and interrupting MYCN through AURKA inhibitor CD532 leads to a notable decrease in tumor burden in patient-derived xenograft mice carrying N-Myc/myrAKT1 tumors.^94^ Notably, CD532 has undergone testing in multiple human cancer cell lines, revealing a robust association between sensitivity to CD532 and the presence of MYCN amplification and expression.^94^ These findings strengthen the case for targeting MYCN, particularly with CD532, as a potential therapeutic strategy in NEPC and other MYCN-amplified malignancies.

## Conclusion and perspectives

Two decades ago, the tantalizing possibility of targeting transcription factors to combat malignancies began to take shape.^183^ Since then, the field has undergone a profound transformation, driven by an exhaustive exploration of protein–protein interactions and the development of cutting-edge techniques for precision targeting.^184^^,^^185^ This evolution has culminated in the clinical targeting of transcription factors, with a particular emphasis on MYCN and the MDM2/p53 axis.

MYCN, a transcription factor with roles not confined to neuroblastoma but extending to various cancer types, presents a multifaceted challenge due to its diverse functions and intricate interactions.^21^ Nevertheless, the research journey has unveiled various innovative strategies to disrupt MYCN-driven tumor development. These strategies encompass the inhibition of MYCN-MAX interactions, the disintegration of super-enhancers, and the modulation of MYCN's transcription and translation. These approaches offer promising avenues and an opportunity to reshape the therapeutic landscape.

As a key survival signaling pathway, the MDM2/p53 axis is widely involved in the development of many tumors. Preclinical and clinical trials provide evidence to support the notion that inhibition of MDM2 could be a potential therapeutic approach for neuroblastoma. The concept of dual-targeted inhibition, which effectively restrains both MYCN and MDM2, along with other critical molecules in neuroblastoma progression, presents an intriguing strategy. Since there is a positive feedback loop between MYCN and MDM2, targeting MDM2 would inhibit both MYCN-mediated tumorigenesis and MDM2-regulated survival of neuroblastoma cells.^28^ Several key directions shape the future of targeting the MYCN-MDM2 pathways in cancer therapy. Innovations in drug delivery systems aim to optimize the precise and efficient delivery of therapeutics to the tumor microenvironment, reducing off-target effects. The exploration of synergistic combination therapies, encompassing targeted agents, immunotherapies, and conventional treatments, remains paramount in achieving maximal therapeutic outcomes while minimizing resistance. Moreover, discovering and validating reliable biomarkers will guide personalized therapy decisions, enhancing patient responses and minimizing adverse effects. Strategies to overcome resistance, a constant challenge in cancer therapy, will be developed and refined. A particular focus will be placed on harnessing the potential of combining MYCN-targeted therapies with complementary modalities to address the complexities of cancer biology more comprehensively. The pursuit of targeting transcription factors like MYCN and the MDM2/p53 axis in cancer therapy has evolved from a theoretical concept to a promising clinical reality. The dynamic and multidisciplinary nature of this field offers hope for more effective and personalized treatments for cancer patients in the future. With continued research, innovative strategies, and a commitment to addressing the complexities of cancer biology, the future holds the promise of transformative breakthroughs in cancer therapy.

## Author contributions

Study concept and design: WW, WL, JF, and RZ; Drafting of the manuscript: WW, YD, and RZ; Revising of the manuscript: WW, SD, FJ, NA, JF, HS, WL, and RZ; Administrative, technical, or material support: WW, WL, and RZ; Study supervision: WW, WL, and RZ. All the authors read and agreed to the published version of the manuscript.

## Funding

This work was supported by the 10.13039/100000002National Institutes of Health (NIH)/National Cancer Institute (No. R01CA214019 to RZ). Additional partial support was provided by the 10.13039/100000054NCI grant R01CA240447 to WL. The content is solely the responsibility of the authors and does not necessarily represent the official views of the National Institutes of Health. WW was supported by a Drug Discovery Institute Seeds Grant.

## Conflict of interests

The authors have no conflict of interests to declare.
